# Shifting trends in influenza and rising HBoV cases: a comparative analysis over 3-winter seasons

**DOI:** 10.1186/s12879-025-12051-6

**Published:** 2025-11-19

**Authors:** Meltem Cetin, Caner Turan, Ceren Yurumez, Ali Yurtseven, Candan Cicek, Eylem Ulas Saz

**Affiliations:** 1 Faculty of Medicine, Department of Pediatrics, Division of Emergency Medicine, Faculty of Medicine, Ege University, Bornova, Izmir, 35100 Turkey; 2 Department of Pediatrics, Ege University Faculty of Medicine, Izmir, Turkey; 3 Department of Clinical Microbiology, Ege University Faculty of Medicine, Izmir, Turkey

**Keywords:** HBoV1, Respiratory infections, RSV, Influenza, Co-infection, Post-pandemic trends

## Abstract

**Background:**

Acute respiratory tract infections (ARTIs) are a major cause of morbidity and hospitalization in pediatric populations. The COVID-19 pandemic has led to significant shifts in the epidemiology of respiratory viruses, particularly *influenza* viruses and *human bocavirus 1* (*HBoV1*). This study aims to investigate the impact of the pandemic on the prevalence of *HBoV1* and its clinical implications in pediatric ARTIs.

**Materials and methods:**

A retrospective cohort study was conducted in a tertiary pediatric emergency department (ED) over three consecutive winter seasons: the pre-pandemic period (2017–2019) and the post-pandemic period (2022–2024). Nasopharyngeal swabs were analyzed using multiplex polymerase chain reaction (PCR) to identify respiratory viruses. Clinical and demographic characteristics, hospitalization rates, disease severity, and outcomes were systematically evaluated.

**Results:**

Among 541 children (median age: 14 months), respiratory viruses were identified in 53.9%. The prevalence of *HBoV1* increased significantly in the post-pandemic period (9.5% vs. 20.8%)(*p < 0.001*), whereas *influenza A/B* infections declined (*p < 0.001*). Co-infections decreased (13.4% to 5.3%), though *HBoV1* was strongly linked to co-infections (*p = 0.001*). Severe illness was significantly associated with co-infections (aOR: 7.3, 95% CI: 3.9–14.2, *p* < 0.001) and age ≤ 1 year (aOR: 2.6, 95% CI: 1.2–5.1, *p* = 0.008).

**Conclusion:**

The post-pandemic period is characterized by an increased prevalence of *HBoV1* and a decline in *influenza A/B* infections. While *HBoV1* alone does not appear to contribute to severe disease, co-infections, particularly with *RSV*, significantly increase morbidity. These findings highlight the need for continued viral surveillance and tailored clinical management strategies to address shifting epidemiological patterns in pediatric ARTIs.

**Clinical trial:**

Not applicable.

## Introduction

Acute respiratory tract infections (ARTIs) are a leading cause of childhood mortality and morbidity, and a common reason for hospitalization [1]. The most common causes of respiratory tract infections are *respiratory syncytial viruses (RSV)*,* Influenza viruses A/B*,* human parainfluenza viruses*,* human rhinoviruses* and *human adenoviruses*. However, in some cases, the causative agent cannot be identified [2, 3]. The use of molecular methods and genomic amplification techniques has enabled the detection of other viruses in etiology, such as *human metapneumovirus*,* human coronavirus*, and *human Bocavirus 1 (HBoV1)* [4].

The Coronavirus disease 2019 (COVID-19) pandemic, officially declared on March 11, 2020, has precipitated substantial alterations in the global epidemiology of respiratory infections [5]. The implementation of public health measures—such as lockdowns, the use of face masks, and enhanced hygiene practices—has led to a temporary reduction in the incidence of seasonal ARTIs. Empirical studies conducted in South Korea and China have documented a decline in *HBoV1* infections during the early phases of the pandemic; however, the prevalence of *HBoV1* remained elevated compared to previous years [6–9]. *HBoV1*, a recently identified viral pathogen, is frequently associated with co-infections and respiratory illnesses in children.

This study aims to systematically examine the alterations in the prevalence of *HBoV1* within pediatric populations following the pandemic and to elucidate its clinical significance in the context of ARTIs.

## Materials and methods

### Study design and setting

**Fig. 1:**
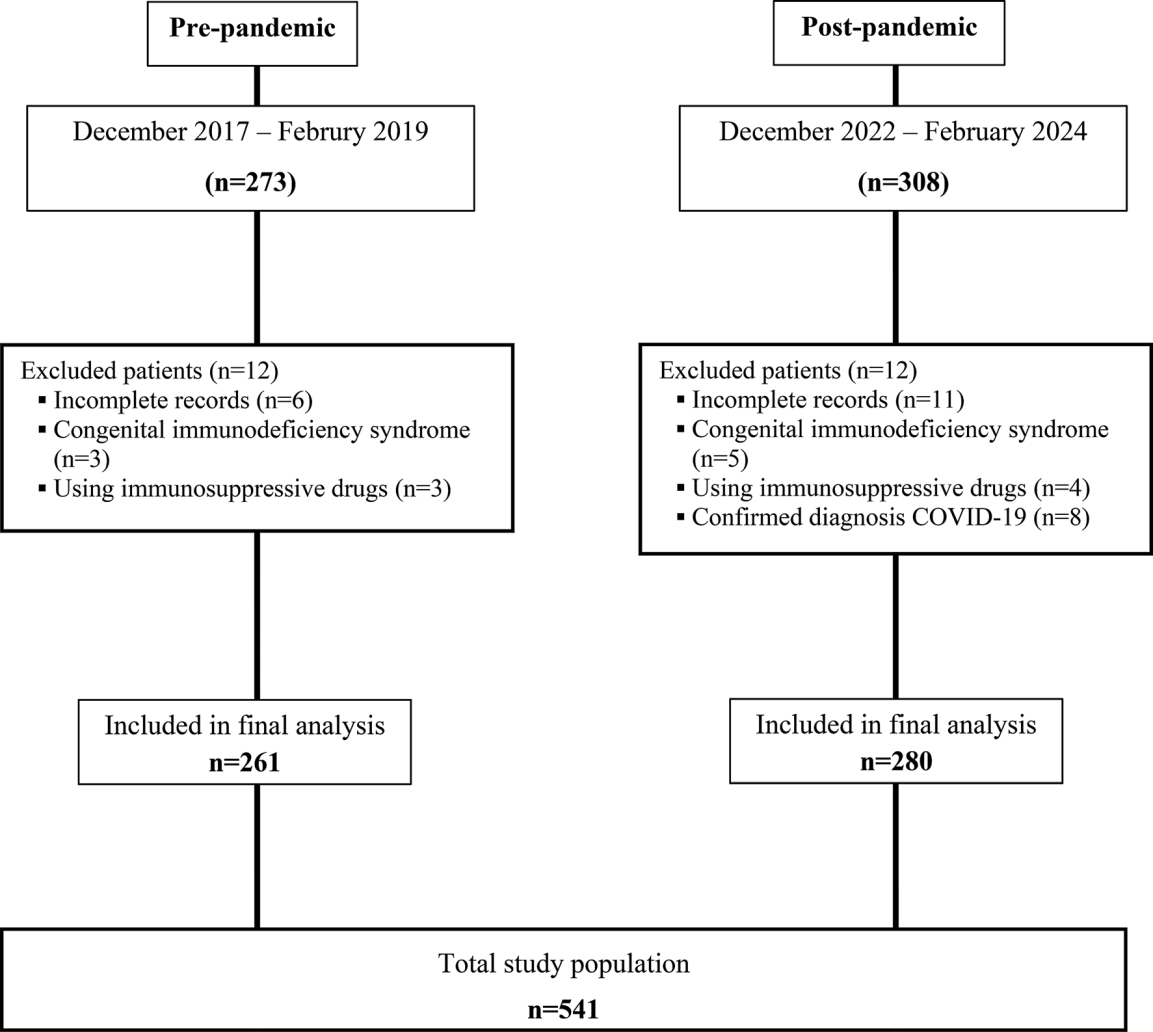
Distribution of patients enrolled in the study period. COVID-19: coronavirus disease 2019

This retrospective study was conducted in the Pediatric Emergency Department (ED) and the Clinical Microbiology Laboratory of Ege University Faculty of Medicine Hospital, a tertiary care center during three distrinct winter periods, the pre-pandemic phase (December-January-February 2017 to 2019) and the post-pandemic phase (December-January-February 2022 to 2024) (Fig. 1). The ED is an academic tertiary care center with approximately 90.000 visits annually. Ethical approval was waived by the local Ethics Committee of Ege University Faculty of Medicine (No. 22-5T/71) in view of the retrospective nature of the study, and all the procedures being performed were part of routine care. The study was conducted in accordance with the Declaration of Helsinki. The data are anonymous, and the requirement for informed consent was therefore waived.

### Patient selection

The study population comprised children aged 0 to 5 years presenting to the ED with respiratory tract infections who underwent respiratory virus multiplex polymerase chain reaction (PCR) testing via nasopharyngeal swab (NPS). Exclusion criteria included children with congenital immunodeficiency syndromes, those receiving immunosuppressive therapy, patients with a confirmed diagnosis of COVID-19 and individuals with incomplete medical records, as illustrated in Fig. 1.

### Clinical algorithm for multiplex PCR testing

The Pediatric ED employs a structured clinical algorithm for the testing of respiratory viruses, which is initiated based on clinical necessity as determined by a pediatric emergency specialist. Indications for multiplex PCR testing include the presence of symptoms consistent with viral respiratory infections, such as cough, nasal congestion or discharge, and coryza. Additionally, testing is indicated in the absence of clinical findings suggestive of bacterial infection, including a toxic appearance, acute sinusitis, or elevated acute phase reactants.

### Data collection and evaluation of patients

The patients’ demographic data, including gender and age, were analyzed along with their main complaints and clinical characteristics upon admission to the ED. PCR-detected respiratory viruses, co-infections, hospitalization [pediatric ward or pediatric intensive care unit (PICU)], length of hospital stay (LOS), and outcomes were recorded. Severe illness is defined as acute respiratory failure and/or the need for non-invasive or invasive ventilation support.

### Respiratory viruses multiplex PCR

The NPS tip is inserted into the nostril and slowly advanced toward the nasopharynx until resistance is felt. Samples are collected by rotating the swab against the nasal wall in a circular motion at least four times. The NPS samples collected from the patients using VTM (Viral Transport Medium) from Copan Diagnostics were sent to the laboratory and analyzed for respiratory viruses using a multiplex PCR test (FTD Respiratory Pathogens 21, Fast-Track Diagnostics, Luxembourg). All samples were kept at 4 °C until submitted to laboratory (within 2 h of sampling). The FTD Respiratory pathogens 21 assay is a qualitative in vitro nucleic acid amplification test for the detection and differentiation of specific viral and bacterial nucleic acids in NPS specimens of human origin. The following respiratory viruses were detected by PCR: *influenza A virus*,* influenza B virus*,* human rhinovirus*,* human coronaviruses (229E*,* NL63*,* HKU1*, and *OC43)*, *human parainfluenza viruses 1–4*,* human metapneumoviruses A* and *B*,* HBoV1*,* RSV A* and *B*,* human parechovirus*,* enterovirus*, and *human adenovirus*.

### Statistical analysis

Statistical analyses were performed using SPSS version 25.0 (IBM, Armonk, NY: IBM Corp.). Continuous variables were expressed as mean ± standard deviation or median (interquartile range), while categorical variables were summarized as counts and percentages. Group differences were evaluated using One-Way ANOVA or the Kruskal-Wallis test for continuous variables, and the Chi-square test for categorical variables. Univariate logistic regression was first conducted to identify potential predictors of severe illness. Variables with a p-value < 0.05 in univariate analysis and clinically relevant factors (age, gender, comorbidity, and study period) were included in the multivariable logistic regression model to calculate adjusted odds ratios (aOR) with 95% confidence intervals. A p-value of less than 0.05 was considered statistically significant.

## Results

### Demographics

A total of 541 children were included in this study, with 48.4% (*n* = 261) of participants in the pre-pandemic phase and 51.6% (*n* = 280) in the post-pandemic phase (Fig. 1). Among the 292 children (53.9%) who tested positive for respiratory viruses, the median age was 14.0 months (IQR 4–48 months). Notably, 29.9% (162/541) of the cohort were aged ≤ 1 year, and the male population constituted 55.2% (299/541) of the total sample. The three most frequently reported symptoms among the participants were cough (64.1%), respiratory distress (55.8%), and nasal congestion/discharge (53.6%) (Table 1).

**Table 1:** Demographic features, complaints and NPS PCR results of patients

Age (m) [median (IQR)]	14.0 (4.0–48.0)
**Age group** (year) (n, *%*)	
>1 ≤ 1	379 *(70.1)* 162 *(29.9)*
**Gender ratio** (M/F)	1.24 / 1
**Admission time** (n, *%*)	
Pre-pandemic Post-pandemic	262 *(48.4)* 279 *(51.6)*
**Complaints** (n, *%*)	
Cough Respiratory Distress Nasal Congestion/Discharge Fever Poor feeding Vomiting	347 *(64.1)* 302 *(55.8)* 290 *(53.6)* 221 *(40.9)* 149 *(27.5)* 51 *(9.4)*
**NPS PCR** (n, *%*)	
+ -	292 *(53.9)* 249 *(46.1)*

F: female, IQR: interquantile range, m: month, M: male, NPS: nasopharyngeal swab, PCR: polymerase chain reaction

### Multiplex PCR

The respiratory viruses most commonly detected were *RSV*,* Influenza A/B*,* rhinovirus*, and *HBoV1* before and after the pandemic, as shown in Table 2. The prevalance of *HBoV1* increased significantly from 15.4% to 20.8% (*p < 0.001*), while Influenza A/B prevalence decreased (*p < 0.001*) in the post-pandemic phase compared to the pre-pandemic.

**Table 2:** The etiology of artis and the frequency of co-infection detected before and after the pandemic

	Pre-pandemic (*n* = 262)	Post-pandemic (*n* = 279)	*p*
**Respiratory virus** (n, *%*)	143 *(54.6)*	149 *(53.4)*	
* RSV* * Influenza A/B* * Rhinovirus* * HBoV1* * HCoV* Other	57 *(21.8)* 52 *(19.8)* 28 *(10.7)* 25 *(9.5)* 5 *(1.9)* 11 *(4.2)*	59 *(21.1)* 3 *(1.1)* 31 *(11.1)* 58 *(20.8)* 8 *(2.9)* 5 *(1.8)*	*0.863* ***< 0.001*** *0.874* ***< 0.001*** *0.467* *0.098*
**Co-infection of viruses** (n, *%*)	35 *(13.4)*	15 *(5.3)*	
2 ≥3	29 *(11.1)* 6 *(2.3)*	11 *(3.9)* 4 *(1.4)*	***0.001***
* HBoV1* + 1 * HBoV1* + ≥ 2	18 *(6.9)* 5 *(1.9)*	7 *(2.5)* 1 *(0.4)*	***0.003***

ARTI: acute respiratory tract infection, HBoV1: human Bocavirus, HCOV: human coronavirus, RSV: Respiratory syncytial virus

Additionally, other respiratory viruses were detected in 4.2% and 1.8% of cases before and after the pandemic, respectively (Table 2). The study found that the prevalence of co-infection among patients was 13.4% (*n* = 35) and 5.3% (*n* = 15) pre-pandemic and post-pandemic phase, respectively (*p = 0.001*). The frequency of co-infection with *HBoV1* was 8.8% (*n* = 23) and 2.9% (*n* = 8) pre-pandemic and post-pandemic phase, respectively (*p = 0.003*) (Table 2). The prevalence of *HBoV1* increased among children who present to the ED with ARTIs in the post-pandemic period compared to the pre-pandemic period. In contrast, there was a notable decrease in the prevalence of *influenza A/B* (*p < 0.001* and *p* < 0.001, respectively) (Table 2).

In the post-pandemic period, the incidence of ARTI with multiple viruses was lower than in the pre-pandemic period. Furthermore, *HBoV1* was detected in 65.7% of co-infections, with a lower frequency of co-occurrence in the post-pandemic period compared to the pre-pandemic period (*p = 0.003*) (Table 2).

### Severity of infections

Despite the increase in *HBoV1* prevalence after the pandemic, the presence of *HBoV1* did not significantly affect the rates of acute respiratory failure and/or the need for non-invasive and/or invasive ventilation (*p = 0.982*). Both univariate and multivariable logistic regression analyses were conducted to identify predictors of severe illness. In univariate analysis, younger age (≤ 1 year), *RSV* positivity, presence of comorbidities, and viral co-infection were significantly associated with severe illness. After adjustment for gender, comorbidities, and study period, age ≤ 1 year (aOR: 2.6, 95% CI: 1.2–5.1, *p* = 0.008), *RSV* infection (aOR: 4.8, 95% CI: 2.8–8.1, *p* < 0.001), and co-infection (aOR: 7.3, 95% CI: 3.9–14.2, *p* < 0.001) remained independent risk factors for severe disease (Table 3). In this cohort, severe illness was observed in 24.5% of the subjects (*n* = 133), manifested by the necessity for advanced respiratory support techniques such as heated humidified high-flow nasal cannula (HHHFNC) oxygen therapy in 11.5% of cases, bilevel positive airway pressure (BIPAP) in 7.4%, and endotracheal intubation in 5.7% of instances (Table 4).

**Table 3:** Predictor factors of severity illness on the admission to the ED

	Severe Illness (+/-)	Univariate OR (95% CI)	*p*	aOR (95% CI)	*p*
**Age** (year)					
≤ 1 >1 *(R)*	74/88 59/320	2.8 (1.4–5.3)	***0.003***	2.6 (1.2–5.1)	***0.008***
**Gender**					
Male Female *(R)*		1.2 (0.8–1.9)	*0.38*	1.1 (0.7–1.8)	*0.51*
**Comorbidity**					
Yes No *(R)*		2.4 (1.1–5.2)	***0.031***	2.1 (1.0–4.6)	***0.045***
**RSV**					
+ ** -** *(R)*	82/34 51/374	5.9 (3.4–9.8)	***< 0.001***	4.8 (2.8–8.1)	***< 0.001***
**HBoV1** + - *(R)*	35/48 98/360	1.0 (0.6–1.8)	*0.95*	0.9 (0.5–1.7)	*0.88*
**Co-infection**					
+ - *(R)*	32/18 101/390	8.6 (4.4–16.1)	***< 0.001***	7.3 (3.9–14.2)	***< 0.001***
**Study period**					
Post-pandemic Pre-pandemic *(R)*		1.4 (0.9–2.2)	*0.11*	1.2 (0.8–2.0)	*0.24*

aOR: adjusted odds ratio, CI: confidence interval, HBoV1: human bocavirus type 1, OR: odds ratio, R: reference, RSV: respiratory syncytial virus

Adjustment variables: age, gender, comorbidity, and study period (pre- vs. post-pandemic)

**Table 4:** Outcomes, interventions and admission features of patients in the ED

**Severe illness (n, %)**	
+	133 *(24.5)*
-	408 *(75.5)*
**Interventions** (n, *%*)	
HHHFNC BiPAP EI	62 *(11.5)* 40 *(7.4)* 31 *(5.7)*
**LOS** [median *(IQR)*]	
ED (h) Hospital (d)	11.5 *(5.0–36.7)* 5 *(3–14)*
**Outcomes** (n, *%*)	
Discharged Ward PICU	251 *(46.4)* 239 *(44.2)* 51 *(9.4)*
**Re-admission in the 24 h** (n, *%*)	
+ -	115 *(21.3)* 426 *(78.7)*

BiPAP: bilevel positive airway pressure, d: days, ED: emergency department, EI: endotracheal intubation, h: hours, HHHFNC: heated humified high-flow nasal cannula, IQR: interquantile range, LOS: length of stay

### Outcomes

After evaluation in the ED, 46.4% of the patients (*n* = 251) were discharged, while 44.2% (*n* = 239) were admitted to the general ward and 9.4% (*n* = 51) were admitted to the PICU (Table 4). Of those discharged from the ED, 21.3% (*n* = 115) returned for further evaluation within the first 24 h for further evaluation. The median LOS in the pediatric ED was recorded at 11.5 h (IQR 5–36.7 h). The median length of hospitalization was reported as 5 days, with an IQR from 3 to 14 days (Table 4).

## Discussion

The findings of this study highlight significant shifts in the prevalence of respiratory viruses in pediatric ARTIs following the COVID-19 pandemic. While multiplex PCR has been reported to detect respiratory viruses in a range of 48–92% of cases [10–12]. The current study observed a lower detection rate of 53.9%. This discrepancy may be attributed to challenges in specimen procurement, variations in assay sensitivity, and the inherent variability associated with pediatric respiratory infections. These results emphasize the complexity of respiratory viral patterns in children, which require careful consideration in clinical settings.

The detection rates of multiplex PCR in children with ARTIs have been reported to range from 48% to 92%, depending on the clinical inclusion criteria, assay sensitivity, number and selection of tested viruses [10–12]. The increase in *HBoV1* prevalence, alongside a decrease in *influenza A/B* infections, suggests a potential alteration in the respiratory viral landscape due to the pandemic’s impact on viral transmission dynamics. This phenomenon aligns with observations from various studies indicating that the COVID-19 pandemic has led to a marked reduction in the incidence of many respiratory viruses, likely due to enhanced public health measures such as mask-wearing and social distancing [13, 14]. For instance, a study conducted in Singapore noted that infection control measures not only curtailed COVID-19 transmission but also reduced the incidence of other respiratory viruses, including *rhinovirus* and *RSV* [13]. This reduction in co-infections is critical as it has been associated with more severe clinical outcomes in pediatric patients [14]. The study’s findings that co-infections were significantly correlated with increased severity of illness underscore the importance of monitoring viral co-infections in pediatric populations, particularly in the context of respiratory illnesses [14]. 

Respiratory tract infections caused by *HBoV1* usually present mild symptoms [15]. However, severe and life-threatening respiratory tract infections have been documented in children experiencing acute respiratory distress [16–18]. The severity of infections observed in this cohort, particularly in children under one year of age, highlights the vulnerability of this demographic to severe respiratory illnesses. The increased risk of severe disease in younger children has been documented in other studies, which emphasize the need for vigilant clinical assessment and management strategies for this group [19]. Interestingly, despite the rise in *HBoV1* prevalence, the study found no significant increase in the need for advanced respiratory support among *HBoV1*-positive patients, suggesting that while *HBoV1* is becoming more prevalent, it may not be associated with increased severity compared to other viruses like *RSV.* [14, 20]

In conclusion, the post-pandemic landscape of pediatric ARTIs is characterized by a notable increase in *HBoV1* prevalence, a decrease in influenza infections, and a reduction in co-infections. These findings have significant implications for clinical practice and public health strategies, highlighting the need for ongoing surveillance of respiratory viruses to inform treatment protocols and preventive measures in pediatric populations. Future studies should aim to elucidate the mechanisms underlying these changes and their potential long-term impacts on respiratory health in children.

The limitations of the study include (1) it was executed within a single tertiary pediatric ED, which may limit the generalizability of the results, (2) due to the retrospective design of this research, data on bacterial PCR analysis were not electronically accessible, and the dataset was confined to information on positive PCR tests only. The retrospective nature of the study also resulted in missing patient data, further complicating the analysis. Consequently, additional research is warranted to elucidate the interactions between *HBoV* and other viral pathogens more comprehensively.

## Conclusion

The COVID-19 pandemic has reshaped the landscape of respiratory viral infections in children, resulting in an increased prevalence of *HBoV1* alongside a concurrent reduction in *Influenza A/B* and co-infections. While *HBoV1* appears to be less associated with severe outcomes when present alone, co-infections, particularly with *RSV*, significantly increase the risk of severe illness and the need for critical care. These findings highlight the necessity for ongoing viral surveillance, updated diagnostic protocols, and tailored clinical management strategies to address the evolving patterns of respiratory infections in pediatric populations. Future longitudinal, multicenter investigations encompassing year-round sampling are warranted to elucidate the temporal patterns and pathogenic significance of *HBoV1*. Clinicians should maintain a high index of suspicion for viral co-infections in young children, as such interactions may influence disease severity, therapeutic decisions, and prognosis.

## Data Availability

The data that support the findings of this study are available from the corresponding author upon reasonable request.
